# The Future of the Creche

**Published:** 1920-11-20

**Authors:** 


### The Future of the Creche.

The secretary to the Council of City Crèches has notified the Hull Corporation that it may not be possible to continue the work unless the Corporation takes over the two crèches. The Corporation have declined to do so, having come to this decision as a result of a report of the medical officer of health, who states that there has been much difference of opinion as to the policy of establishing crèches or day nurseries for children under school age. Voluntary organisations such as the Council of City Crèches, he says, have done good and useful work, for until now the crèches have been organised entirely by voluntary effort and supported chiefly by means of voluntary subscriptions. While crèches are under voluntary management it is not practicable to make unduly stringent regulations as regards premises, equipment, etc. But under municipal management the Ministry of Health would expect higher standards of efficiency to be maintained. In order to bring about some measure of standardisation, the Ministry of Health recently issued a memorandum in regard to day nurseries, in which is given the minimum standard of efficiency that must be secured in the organisation and conduct of a nursery. Neither of the local crèches comes up to this standard. The medical officer is therefore unable to recommend the taking over of the crèches on the ground that the initial expense of probable alterations and equipment, together with the cost of maintenance, is, in spite of the Government grant of 50 per cent., likely to be so heavy as to outweigh the doubtful benefit of these places.

The whole question of crèches needs far more consideration than it has hitherto received. The obvious advantages are apt in the eyes of sympathisers to obscure their dangers. Carried on in unsuitable premises, and without sufficient means of support to maintain a skilled staff, they may easily do more harm than good. We believe them to be an indispensable aid in certain localities and at the existing stage of social development, and regret that the Hull Corporation have declined to assume responsibility.

